# Drawing cancer chronicles: A qualitative study to evaluate narrative meaning-making over time and in response to a meaning-centred care intervention

**DOI:** 10.1371/journal.pone.0341150

**Published:** 2026-01-20

**Authors:** Emily R.E. Evans, Niels van Poecke, Maite Portela, Michael Scherer-Rath, Yvonne Weeseman, Zarah M. Bood, Nirav Christophe, Henny Dörr, Judith de Vos-Geelen, Aart Beeker, Gerty de Klerk, Eric Bras, Hugo Vlug, Frans Bossink, Frans Savelkoul, Liesbeth M. Timmermans, Mirjam A.G. Sprangers, Esther Helmich, Hanneke W.M. van Laarhoven

**Affiliations:** 1 Department of Medical Oncology, Amsterdam University Medical Center, University of Amsterdam, Amsterdam, The Netherlands; 2 Cancer Center Amsterdam, Cancer Treatment and Quality of Life, Amsterdam, The Netherlands; 3 Department of Elderly Care and Research in Education, Amsterdam University Medical Center, Amsterdam, The Netherlands; 4 Department of Philosophy, Theology and Religious Studies, Radboud University Nijmegen, Nijmegen, The Netherlands; 5 iHUB, Rotterdam, The Netherlands; 6 HKU University of the Arts, Utrecht, The Netherlands; 7 Department of Internal Medicine, Division Medical Oncology, GROW – Research Institute for Oncology & Reproduction, Maastricht University Medical Center, Maastricht, The Netherlands; 8 Department of Medical Oncology, Spaarne Gasthuis, Hoofddorp/Haarlem, The Netherlands; 9 Department of Pastoral and Spiritual Care, Amsterdam University Medical Center, location VUmc, Amsterdam, The Netherlands; 10 Department of Pastoral and Spiritual Care, Amsterdam University Medical Center, location AMC, Amsterdam, The Netherlands; 11 Department of Spiritual Care, Spaarne Gasthuis, Haarlem/Hoofddorp, The Netherlands; 12 Department of Spiritual Care, Maastricht University Medical Center, Maastricht, The Netherlands; 13 Department of Primary and Community Care, Radboud University Medical Centre, Nijmegen, The Netherlands; 14 Department of Medical Psychology, Amsterdam University Medical Center, University of Amsterdam, Amsterdam, The Netherlands; 15 Amsta Healthcare Organization, Amsterdam, The Netherlands; Ural Federal University named after the first President of Russia B N Yeltsin Institute of Physics and Technology: Ural'skij federal'nyj universitet imeni pervogo Prezidenta Rossii B N El'cina Fiziko-tehnologiceskij institut, RUSSIAN FEDERATION

## Abstract

The diagnosis of incurable cancer can disrupt life stories, undermining meaning-making and challenging self-identity. People may therefore need to search for and create new stories about their lives that incorporate their diagnosis. Arts-based narrative interventions are being explored to support this existential process of narrative meaning-making. However, developing effective existential support may be limited by the lack of methods capable of investigating their impact. This study is the first to explore narrative meaning-making across time and a meaning-centred care intervention by longitudinally using ‘Rich Pictures’ (RPs) – hand-drawn visual narratives. We analysed repeated RPs about living with incurable cancer from thirty-eight participants in two studies: one incorporating an arts-based narrative intervention, and one without. RPs were compared across time and the two groups using an inductive, multi-level, and participatory analysis approach. Our findings highlighted six strategies variably used by participants to reconstruct their narratives over time: repeating, retaining, repurposing, reinforcing, reducing, and reassembling. Differences were found in the employment of these strategies between the two different studies, with arts-based intervention participants predominantly developing new ways of narrating and relating to cancer as a disruptive life event. We conclude that people living with incurable cancer employ a range of strategies in reconstructing their narratives. Arts-based interventions may support this existential process. The repeated use of RPs is a valuable method for investigating narrative meaning-making over time, across groups, and interventions, offering insights to evaluate and develop meaning-centred care in oncology.

## Introduction

Despite advances in oncological treatment and palliative care, people living with incurable cancer often face profound existential distress [1–3]. However, current care frameworks often fail to support existential processes adequately [1,4–6]. While clinical care increasingly focuses on quality of life and shared decision-making, the deeper question of how individuals make sense of their altered lives remains underexplored. Addressing this need requires attention to the way people construct meaning, identity, and coherence in the face of serious illness.

A key aspect of this process lies in the stories we construct about ourselves and our experiences. These narratives play a crucial role in life, helping us understand the world around us, find meaning in our experiences, and establish a sense of who we are – an existential process defined as ‘narrative meaning-making’ [3,7]. These stories are neither fixed nor finalized, but rather evolving, reconstructed, and retold as life unfolds over time [8–12].

Reconstructing these stories may become especially significant as people are confronted with unexpected, life-altering events – such as the diagnosis of incurable cancer [3,8]. Such events may evoke an ‘experience of contingency’: a profound disruption to the existing life story that gives rise to existential concerns by undermining the ability to find meaning and challenging self-identity [3,8]. As a result, people living with incurable cancer may need to fundamentally rethink their life stories and construct new narratives that integrate their diagnosis [3,8].

Constructing stories about living with incurable cancer is not an entirely individual process. It is a ‘collaborative enterprise’ involving the co-construction of narratives in interactions with others, storytelling conventions, and available resources – including the arts [9,11–15]. Storytelling, and the life stories told, are thus conceptualized as both deeply personal and inherently social [9,13–15]. Because stories are told to others within particular social and cultural settings, the process of narrating one’s life is not entirely free but shaped – and sometimes limited – by the expectations, language, resources, and norms available within those contexts [9,10,16].

In particular, verbal narrative conventions may generate constrained illness stories by failing to offer the appropriate ways and words for the articulation of illness experiences [17]. Alternatively, interactions with arts-based resources such as literature, music, and visual art, have been increasingly explored as ways to support the reconstruction of illness stories [11]. Within a supportive care context targeting unmet existential needs, this has led to the development of arts-based narrative interventions supporting narrative meaning-making by people living with incurable cancer [17–20].

However, developing effective (arts-based narrative) interventions within care contexts that address existential concerns may be limited by the availability of methods for exploring existential processes [1,5,6]. Whilst questionnaires (e.g., [21]) and interviews are valuable, they may fall short in depth of insights required to research changes at an existential level [6]. As a result, calls have been made to investigate narrative outcomes by employing different methods [6]. One promising approach emerging in illness narrative research is the use of so-called ‘Rich Pictures’ (RPs) – hand-drawn, visual narratives that depict complex issues, experiences or domains in a single, holistic image [22, 23]. RPs have been shown to tell multidimensional illness stories in a comprehensive ‘snapshot’ [24]. The longitudinal use of RPs may be a potentially unique approach for investigating the process by which individuals (are able to) reconstruct their narratives of illness, both over time and in response to care interventions [25]. The insights RPs might offer in this context, however, remain largely unexplored. This study therefore explores (a) the reconstruction of narratives about living with incurable cancer over time; and (b) the potential support offered by an arts-based narrative intervention in this process, by using RPs.

## Method

We analysed RPs from two distinct studies, each involving repeated RP creation over time. The first study, ‘In Search of Stories’ (ISOS), implemented an arts-based narrative intervention, with RPs drawn at its beginning and end [18–20,26]. The second study was a preparatory project for ISOS, piloting the repeated use of RPs over time, without further intervention [24,25]. This group is thus referred to as the ‘No Intervention’ (NI) group. Together, these studies provided a longitudinal corpus of RPs (ISOS RP1 and ISOS RP2; NI RP1 and NI RP2) available for the current ‘Drawing Cancer Chronicles’ (DCC) analyses.

### Drawing cancer chronicles recruitment

Eligibility and recruitment for the included ISOS and NI studies have been described elsewhere [20,24,25]. In brief, inclusion criteria were: a diagnosis of incurable cancer (any solid tumour type); age ≥ 18 years; sufficient mental, verbal and physical capacity to participate; and written informed consent. ISOS had additional requirements of a life expectancy ≥ proposed 6-month duration of the intervention; and exclusion criteria of a WHO performance of ≥3 or a Karnofsky performance score of <60, and inability to speak Dutch [20]. Recruitment took place between 1^st^ February 2018 and 1^st^ May 2018 (NI); and 1^st^ March 2021 and 1^st^ August 2022 (ISOS).

For the DCC analyses, ISOS participants were included in the RP1 analyses if they had completed the first RP. For comparative analyses, only those who completed both RP1 and RP2 were included. NI participants were required to have completed both RP1 and RP2 to be included in any DCC analyses, as their data was specifically used to analyse narrative changes over time compared to the intervention group.

### Drawing repeated rich pictures in the ISOS and NI studies

**Fig 1:**
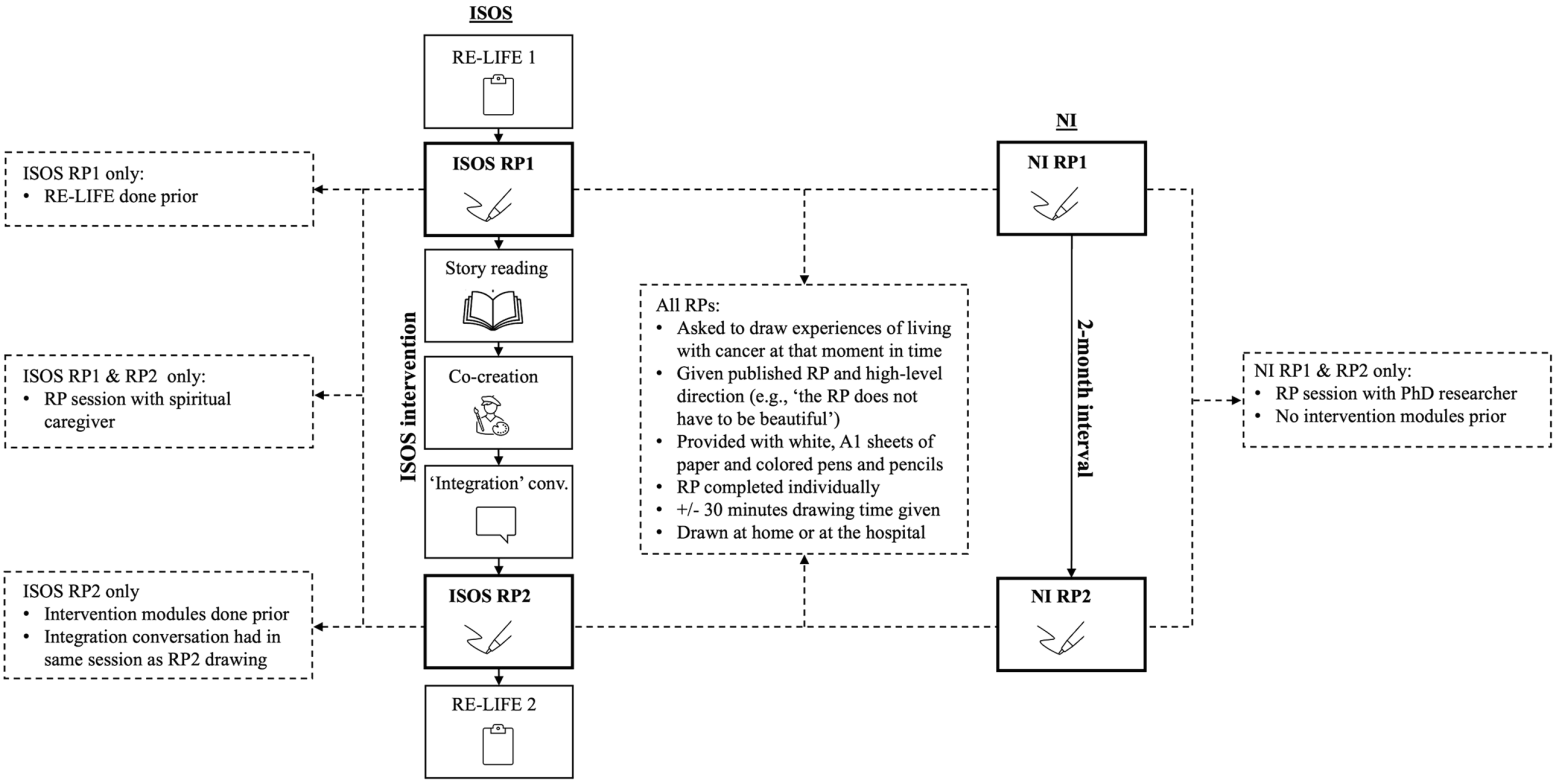
Drawing repeated Rich Pictures in the ISOS and NI studies. This figure depicts the overall processes for RP creation within the two ISOS and NI studies, and their relative convergences and divergences. The stepwise process shown on the inner-left of the figure depicts the ISOS intervention trajectory and the sequence of its modalities. The two-step process shown on the inner-right depicts the NI study trajectory and its two-month interval, without further intervention. Points at which RP1 and RP2 creation occurred within these trajectories are shown in bold. The dashed box in the centre of the figure, connecting all ISOS and NI RPs, highlights all convergences between and within the two study designs for drawing RPs. Dashed boxes on the outer-left and -right highlight divergences between the ISOS and NI study designs for RP drawing. Within these, dashed boxes on the outer-left, and positioned vertically at the top and bottom, indicate project-specific divergences between ISOS RP1 and ISOS RP2 drawing. Dashed boxes on both the outer-left and -right, and positioned central-vertically, highlight convergences between project-specific RP drawing, but divergences across the two projects.

ISOS participants were invited to draw two RPs: one at the beginning and one at the end of the ISOS intervention (see Fig 1). Prior to RP1, ISOS participants completed and discussed a ‘Reconstruction of Life Events’ (RE-LIFE) questionnaire [21]. At the time of drawing RP2, they had completed the co-creation process with artists, engaged with selected literature, and had an ‘integration’ conversation with a spiritual caregiver reflecting on the ISOS process and their life story [18–20]. No other psychosocial interventions were provided within the project, though participants could seek further support elsewhere [20]. NI participants were invited to draw two RPs with a two-month interval and no further study intervention (see Fig 1) [25].

All participants drew their RPs individually during in-person sessions with a spiritual caregiver (ISOS: EB, HV, FB, FS), or PhD candidate (NI: ZB). Spiritual caregivers were instructed on the RP method and provided with a protocol. The PhD candidate was trained by an experienced RP researcher (EH) [24,25]. RP sessions were held at the hospital or participant’s home [25].

All participants were asked to draw a RP about their experiences of living with cancer at that moment in time [25]. White A1 paper and coloured pens and pencils were provided. Participants were given the same published RP example [27] and high-level direction, e.g., ‘the rich picture does not have to be beautiful’, useful when inviting RP creation [22,25,28]. All participants were left alone to draw for approximately 30 minutes. Participants were then interviewed about their RP. In the RP2 session, participants could choose whether to see their RP1 and whether to continue with it or draw a new RP. For ISOS participants, this session also included the integration conversation.

### Demographic and clinical data

Demographic and clinical data were collected from the electronic patient files by the research team (ZB, NvP, YW), and/or attending oncologist (e.g., HvL, AB, JdVG, GdK). Additional data for ISOS participants, including educational level and occupation, was collected through a ‘General Questionnaire’.

### Analysing rich pictures

Researchers analysing RPs place different emphasis on RPs themselves and interviews about them. Whilst analysis approaches prioritising the interviews are valuable, they may overlook insights RPs themselves can make to narrative research [29–34]. We therefore analysed the RPs as visual narratives about life with cancer, without interviews, aiming to compare them over time and across groups. To do this, we conducted an inductive, multi-level, and participatory analysis process. This is described in detail elsewhere and summarised below [34].

MAXQDA (version 22.1.1) was used in the analysis. Analysis was primarily conducted by EE – a female PhD candidate in participatory arts-based research in oncology with a background in art and sociology. Analysis, including its consistency, was supported by a second coder (MP) and through regular discussions together with the core research team (EH, HvL, NvP). Backgrounds here were interdisciplinary, including medical education, elderly care, qualitative and RP research, sociology of health and illness, cultural sociology, medical oncology, aesthetics, and theology.

#### Multi-level analysis of rich pictures.

The multi-level analysis process for the RPs consisted of three levels: (1) a ‘core analysis’, (2) a ‘timepoint-to-timepoint comparison’ (T-T), and (3) an ‘inter-group comparison’ [34]. The ‘core analysis’ was a systematic inductive coding process exploring participants’ visual narratives at a single timepoint. This process moved from icon-by-icon, in vivo coding, through descriptive and interpretative coding, to the inductive development of broader narrative categories within each RP subset. This level was the foundation for our comparative analyses. The ‘timepoint-to-timepoint comparison’ was an inductive, comparative analysis process focused on repeated RPs drawn over time – comparing ISOS RP1s with ISOS RP2s, and NI RP1s with NI RP2s. Visual and core analysis-assisted comparisons of RP-pairs generated descriptive comparative codes, then organized into preliminary comparative categories. Analysis then shifted to developing major comparative categories across participants. Through this process, we inductively developed the narrative strategies discussed hereafter. Finally, the ‘inter-group comparison’ compared repeated ISOS RPs with the repeated NI RPs, leveraging all previous analyses to explore the (way) narrative categories and strategies (were) appearing across the two datasets.

#### Participatory analysis of rich pictures.

Analysis was supported by so-called ‘gallery walks’ – a participatory analysis method often used in RP research. In this approach, multiple RPs were viewed and discussed by different groups of participants [22,24,34]. Gallery walks were used to address the polysemy – possibility for multiple meanings – and polyvocality – possibility for multiple voices and perspectives – when interpreting visuals. Through exploring various interpretations in the gallery walks, we aimed to develop rich and intersubjective understandings of the RPs [22,34]. This process informed the development of our coding and helped to incorporate triangulation, reflexivity, and co-creation into our methodology.

Our analysis included two gallery walks (GW1 and GW2). GW1 was designed for an interdisciplinary group of researchers offering different academic perspectives, with inclusion criteria of age ≥ 18 years; academic expertise that may be relevant to RP interpretation; and oral informed consent. GW2 was designed for people from the same illness group, i.e., people with incurable cancer, and (in)formal caregivers, offering different lived and clinical perspectives. GW2 inclusion criteria were: age ≥ 18 years, fluency in Dutch, and written informed consent; as well as one of the following: a diagnosis of advanced cancer and sufficiently well to participate (in terms of WHO or Karnofsky performance); cancer patient representative; or (in)formal caregiver. GW1 and GW2 participants were recruited from the Amsterdam University Medical Center, or through our professional networks (HvL, EH, NvP, EE).

Gallery walks took place at the Amsterdam University Medical Center in September 2023 and June 2024, and lasted 75 minutes (GW1) and 90 minutes (GW2), respectively. They were participant-driven, semi-structured sessions involving iterative steps to assist group interpretations and comparisons of the RPs [34]. Sessions were guided by EH and supported by EE. ISOS RP1s and ISOS RP2s were included. Audio and visual recordings, as well as written fieldnotes by EE, were collected [34]. Gallery walks were immediately reflected upon and summarised through dialogue between EE and EH. Fieldnotes immediately informed the coding of the RPs. Visual data helped to relate audio or notes to specific RPs. Audio recordings were analysed inductively in MAXQDA for discursive interpretations of the RPs. Insights were then deductively connected to the pre-existing coding of the RPs, and pre-existing codes deductively explored in the gallery walk audios. New findings were thus integrated and pre-existing insights supported and enriched. Data saturation was achieved overall when no new narrative categories, strategies, and inter-group comparisons arose from the (integrated) multi-level and gallery walk analyses.

Through this process, analyses of the RPs became highly polyvocal, layering and combining interpretations from our research team and gallery walk participants. To acknowledge this polyvocality, gallery walk excerpts are included throughout our findings [33].

### Ethics

The study was exempted from ethical approval by the Medical Ethics Review Committee of the Academic Medical Center and Amsterdam University Medical Center, since the Medical Research Involving Human Subjects Act was not applicable (reference number: W20_436 # 20.483; W17_476 # 17.549; 2024.0319). Written informed consent was obtained at the start of participation from those drawing RPs and included in GW2. Oral informed consent was obtained from academic peers, prior to and at the start of GW1, and is documented through email exchange.

## Results

### Drawing cancer chronicles participant inclusion

Our DCC analyses included thirty-eight participants (ISOS, n = 25; NI, n = 13) (see S1 Fig). We analysed a corpus of sixty-three RPs (ISOS: RP1s, n = 25; RP2s, n = 12; and NI: RP1s, n = 13; RP2s, n = 13).

RP1 analyses included all thirty-eight participants and their corresponding thirty-eight RP1s. RP2 analyses included twenty-five participants (ISOS, n = 12; NI, n = 13) and their twenty-five RP2s. Comparative analyses focused on these twenty-five participants who completed both RP1 and RP2, using their paired RPs (a total of fifty RPs).

### Drawing cancer chronicles participant characteristics

S1 Table presents the characteristics of participants in ISOS and in the NI group [25].

#### ISOS participants.

ISOS participants were predominantly female (n = 18). The age of participants ranged between 28–80 years, with a median age of 59. ISOS participants were mostly theoretically educated (i.e., receiving academic education or attending a university of applied science) (n = 19), and had (very) high skill-level occupations in the top three ISCO-08 Major Categories (n = 20) [35–37].

ISOS participants had diagnoses across twelve different advanced cancer types, most commonly breast cancer (n = 6). WHO performance status at the time of the first RP drawing ranged from 0 to 2, with most ISOS participants reporting 0 (n = 14) or 1 (n = 10). The time between ISOS participants’ diagnosis of advanced cancer and drawing their first RP varied from 3 to 86 months, with a median of 9 months. However, over half of participants had recent disease progression (n = 14), with a median of 1.5 months between this progression and their first RP.

ISOS participants with 2 RPs (n = 12) mostly had the same WHO performance status at the time of drawing their first and second RPs (n = 10). Time between RP1 and RP2 differed between participants, ranging from 4 to 12 months, with a median of 7 months. There are no notable differences between the overall ISOS group and the smaller ISOS group of participants with 2 RPs.

#### NI participants.

NI participants were almost evenly male (n = 7) and female (n = 6). Their ages ranged between 31–79 years, with a median of 64 years. Data for education level was missing (n = 8) as this information is rarely recorded in the electronic patient files. NI participants with 2 RPs had (very) high skill-level occupations within the top two ISCO-08 Major Categories (n = 7), moderate-low skill level occupations within three of the lower four ISCO-08 Major Categories (n = 4), or unknown occupations (n = 2) [35–37].

NI participants had diagnoses across four advanced cancer types: oesophageal cancer (n = 5), pancreatic (n = 4), gastric (n = 3) and colon (n = 1) cancer. They largely had a WHO performance status of 0 (n = 6) and 1 (n = 5) at the time of drawing their first RP. This remained the same for half the NI group when drawing RP2 (n = 6), while for the other half, it either decreased (n = 3) or increased (n = 3).

Time between NI participants’ diagnosis of advanced cancer and first RP varied between 2–45 months, with a median of 21 months. Three NI participants had additional disease progression between 2–28 months prior to their first RP. The duration between drawing the first and second RP was predominantly 2 months (n = 10).

#### Gallery walk participant inclusion and demographics.

Twelve participants were included in the gallery walks, with six participants in each session. GW1 comprised of four PhD candidates and two (associate) professors, with backgrounds in oncology, palliative care, medical education, RP research, graphic medicine, and the medical humanities. All PhD candidates were medical doctors. GW2 included three participants with a diagnosis of incurable cancer, two formal caregivers specialising in palliative care and medical oncology, and one informal caregiver – a partner of a participant with incurable cancer.

### Comparing RPs reveals strategies for narrative reconstruction over time

By comparing RPs over time, six – often co-existing – strategies for the reconstruction of visual narratives about living with incurable cancer over time were found: (1) repeating – the deliberate re-presentation of the same, unchanged visual narrative; (2) retaining – carrying forward aspects of their visual narratives from one RP to the next; (3) repurposing – the adaptation or transformation of previously used narrative aspects; (4) reinforcing – the intensification or amplification of narrative aspects; (5) reducing – the shrinkage or removal of narrative aspects; and finally (6) reassembling – the visual reorganisation or reordering of aspects of, or the whole, RP. Together, these strategies illustrate the dynamic and multifaceted ways participants expressed and adapted their visual illness narratives over time. By comparing the repeated ISOS RPs and the repeated NI RPs, differences in the use of strategies, and (subsequent) changes in their narratives, were observed. Here we present these findings, highlighting each strategy.

#### Repeating.

**Fig 2:**
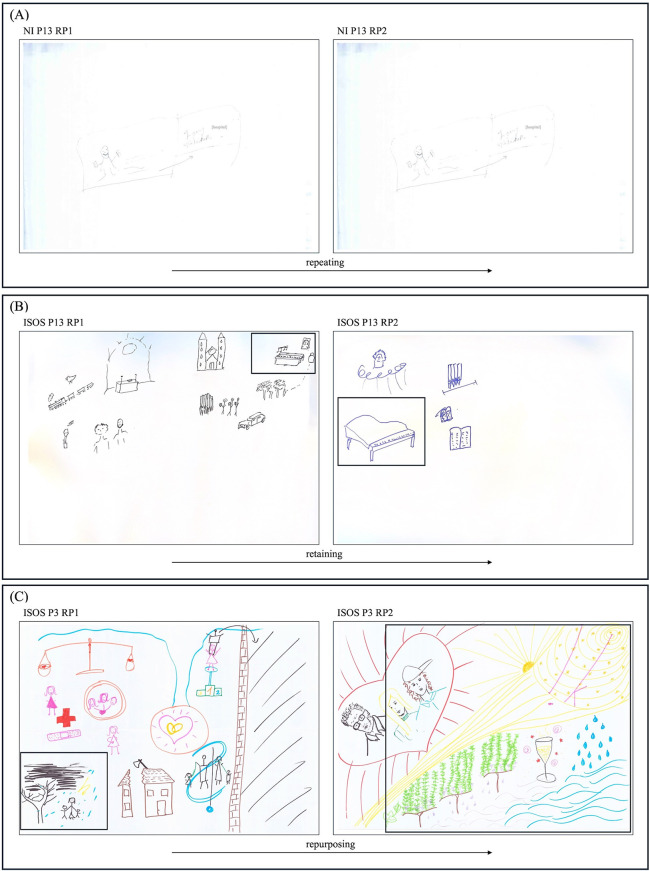
Rich Pictures exemplifying the strategies of repeating, retaining, and repurposing. **(A)** NI P13’s RP exemplifying the strategy of repeating. RP1 depicts a singular scene of travelling in-between the hospital – a common visualisation of transition and being ‘in-between’ in the RPs. By repeating this RP1, NI P13 may narrate the ongoing experience of being durationally suspended in transition. **(B)** ISOS P13’s RP1 and RP2 exemplifying the strategy of retention in relation to meaningful everyday activities. This can be seen in the re-use of the piano drawn in RP1 in RP2. **(C)** ISOS P3’s RP1 and RP2 exemplifying the strategy of repurposing in relation to the use of nature. This can be seen as nature shifts from a metaphor of disruption (e.g., being struck by lightning) to flow (e.g., depictions of flows of water or sunrays).

Few participants chose not to create a second RP, instead reusing and re-telling their unchanged RP1 in the RP2 session (see Fig 2A). Rather than viewing the repetition of RPs as merely unchanged narratives, this recurrence appeared to serve as a meaningful narrative strategy. It reflected participants’ ongoing experiences of liminality – states of suspension, ambiguity, or feeling ‘stuck’ – which were thematically dominant in the content of all the repeated RPs themselves. The absence of narrative progression thus appeared not simply incidental but seemed to mirror the very condition being depicted: a sense of arrested transition or non-movement, expressed both visually and through the repeated use of the same image over time. Given the few participants who used this strategy, gallery walk participants did not comment on the repeated RPs.

#### Retaining.

Distinct from the strategy of repetition, ‘retaining’ referred to a more selective re-use of aspects of one RP in the next, without fully repeating an unchanged RP1 (see Fig 2B). As articulated by the gallery walk participants as they connected the RPs of ISOS P13:

GW1 P1: I thought the piano picture was connected to this one...

GW1 P2: Yeah, yeah, yeah

GW1 P3: Because there is also this piano here

Most ISOS participants, as well as NI participants, used this strategy, making it the most employed approach. Retention varied in degree – from retaining large portions of RP1s to preserving very specific parts – and was seen to occur across all the various aspects of their narratives. Social relations were the most prominently retained aspect of the narratives among both ISOS and NI participants, within which participants primarily focused on retaining social connection and support. Though both groups of participants mostly used this strategy, differences were found between its specific use by ISOS and NI participants. For example, many NI participants appeared to retain aspects of medical and somatic experiences, and themes of disruption. Comparatively, few ISOS participants were seen to do so.

By employing the strategy of retention, ISOS and NI participants may, on the one hand, have worked to maintain narrative continuity between their RPs in aspects particularly important to them, and on the other, with NI participants particularly, highlighted their ongoing (narrative negotiation of their) experiences of disruption.

#### Repurposing.

Whilst retention led to an observable ‘sameness’ of the RPs, retained aspects of RPs were often “not *exactly* the same” (GW2 P2). Indeed, as participants kept aspects of their narratives, their nuances often changed, developing a sense of continuity ‘with a twist’. This strategy came to be denoted as ‘repurposing’ (see Fig 2C). As exemplified by gallery walk participants in discussion on ISOS P25’s RP1 and 2:

GW2 P5: It’s the same

GW2 P3: The exact same

GW2 P2: No, there it says only “holidays” and here there is a caption

GW2 P3: The person who drew this enjoyed going on holiday, and now their holidays have become memories

Participants could often be observed to repurpose social and medical aspects included in their narratives, as well as their use of nature, and meaningful everyday activities. Used by few NI participants, repurposing was, however, used by most ISOS participants. For example, ISOS P3 repurposed their use of nature from a metaphor of disruption to a metaphor of flow (Fig 2C); whilst ISOS P1 and P13 were both seen to retain religious aspects of their drawing, but did not now depict them as religious buildings or spaces – as done in RP1 – but rather as symbols (see also Fig 2B).

Repurposing thus denoted the adaptation or transformation of the same aspects so that they might continue as part of the narrative – even when their original form was perhaps no longer possible or relevant. This highlighted how narrative continuity coexisted with narrative adaptation, as participants repurposed retained aspects as their perspectives and experiences evolved.

#### Reinforcing.

**Fig 3:**
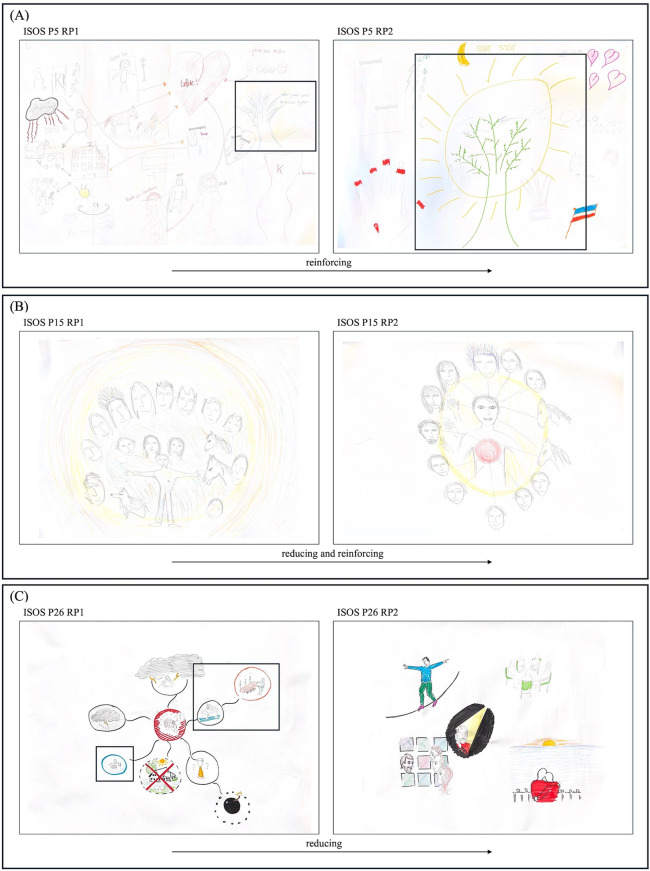
Rich Pictures exemplifying the strategies of reinforcing and reducing. **(A)** ISOS P5’s RPs exemplifying the strategy of reinforcing in relation to positive metaphors of nature. This can be seen as the tree and sun depicted in RP1 are increased size, foregrounded and made central, and depicted with bolder colour and changed drawing style in RP2. **(B)** ISOS P15’s RPs exemplifying the complementarity of reducing and reinforcing in relation to social relations. This can be seen as the surrounding yellow sphere in RP1 becomes smaller in RP2, proximity to self becomes closer, and connections between people are made explicit (see now the connecting lines and hands in RP2 which were not present in RP1). **(C)** ISOS P26’s RPs exemplifying the strategy of reduction.* This can be seen in relation to, for example, medical and somatic aspects, as depictions of treatment and death in RP1, are no longer depicted in RP2. All other disruptions depicted in RP1 (e.g., lightning strikes, bombs, barriers) are also removed in RP2, and the narrative is refined to reduced aspects. Please note that these RPs also serve as an example of the strategy of reassembling. * These RPs, without amendments indicating strategies, were published before under the terms of the Creative Commons Attribution License (CC BY 4.0) [26].

As well as repurposing retained narrative aspects, participants often ‘reinforced’ specific aspects, indicating their intensification (see Fig 3A). This was often depicted through increased size, frequency, or having “more” of a certain narrative aspect, greater foregrounding and centrality, and closer proximity to oneself. This process was also highlighted by gallery walk participants in their discussion:

GW2 P5: There’s more sun. You see it there, there, there, there.

GW2 P6: Yes, precisely. And this tree [ISOS P5]... It is now drawn with such passion.

Additionally, participants reinforced certain aspects of their drawings by way of their drawing style – as articulated by GW2 P6 – or by making them “bold” or “clearer” (GW2 P2) by (re-)using colour. Participants also indicated that new aspects became intensified in their narratives, by adding entirely new ones, or ones within preexisting categories, such as finding new meaningful activities, including the ISOS project itself.

Reinforcing was again a strategy primarily employed by ISOS participants, though some NI participants also used it. Participants largely reinforced depictions of social relations (specifically, social connectivity and support), (positive metaphors of) nature, or meaningful everyday activities. In doing so, this may be interpreted as the active reinforcement of identities beyond their cancer, as described by GW1 participants:

GW1 P3: I think it’s not just “I like” [these everyday activities], it is “I am more than my cancer. I *am* all of these things.”

EH: So, it’s not only “I like playing the piano” [ISOS P13 RP2, Fig 2B] but, “this is my life”! It is central.

Notably, however, some NI participants used reinforcement to intensify themes of disruption in their narratives – a strategy observed to be done by only one ISOS participant. In contrast, ISOS participants were seen to use reinforcement to intensify themes of progression and flow.

#### Reducing.

The strategy of reducing denoted the less frequent use of visual narrative elements, removal, shrinkage in size, crossing out, containment, or backgrounding of visual narrative elements, as well as simplification of drawing style (see Fig 3B and 3C).

GW2 P5: I find this calming because this is [drawn] very separate. Here, only four things are drawn and nothing more. Whereas here [referencing RP1s] many things are drawn – and they are more scribbled.

This strategy appeared to serve a dual function in the reconstruction of participants’ narratives. First, reduction indicated meaningful refinement of participants narratives, diminishing disruptive aspects whilst distilling others – as GW2 P5 describes above – and often creating ‘room’ for the strategy of reinforcement. Reinforcement and reduction were thus often seen to be complementary strategies. Second, and sometimes simultaneously, reduction signalled disruptive loss or rejection. For example, NI P2 reduced their RP to a single metaphor – an ostrich burying its head in the sand – powerfully depicting their experience of withdrawal over time. In discussion about the reduction of social relations (see also Fig 3B), GW2 participants described this dual function through their own social experiences:

GW2 P6: It can be read that they have few good friends, *or* I want less but *good* friends around me. Because I have many friends, but some of them I have had to put on hold for the moment [...].

GW2 P3: You have a need for a smaller circle. I recognise this. I have several friends who cannot cope with the situation, so we have a lot of difficulty having contact with one another. I know it is because of their ignorance or inability – maybe this isn’t the right word. They are also searching. I find this difficult. You miss the conversation. You miss the contact.

Reduction was seen across all aspects of the narratives, and again to varying degrees, with some participants reducing their entire RPs to a single scene and some crossing out minor aspects. This strategy was used by many ISOS participants, and some NI participants. However, a notable difference between these groups could again be observed when it came to themes of disruption. Only ISOS participants – and thus not the NI group participants – fully removed disruption from their narratives; with all remaining ISOS participants who did not eliminate disruption entirely, able to reduce it. Yet comparatively, few NI participants were able to even shrink or contain disruption. When discussing the ISOS RPs, the gallery walk participants highlighted this, along with the complementarity of reduction and reinforcement as distinct narrative strategies:

GW2 P6: I find that in general there is more of a sense of peace... Joy sometimes too, I think. […] I see it in the colour... Bold drawings... The coffins are missing. They have totally gone.

#### Reassembling.

**Fig 4:**
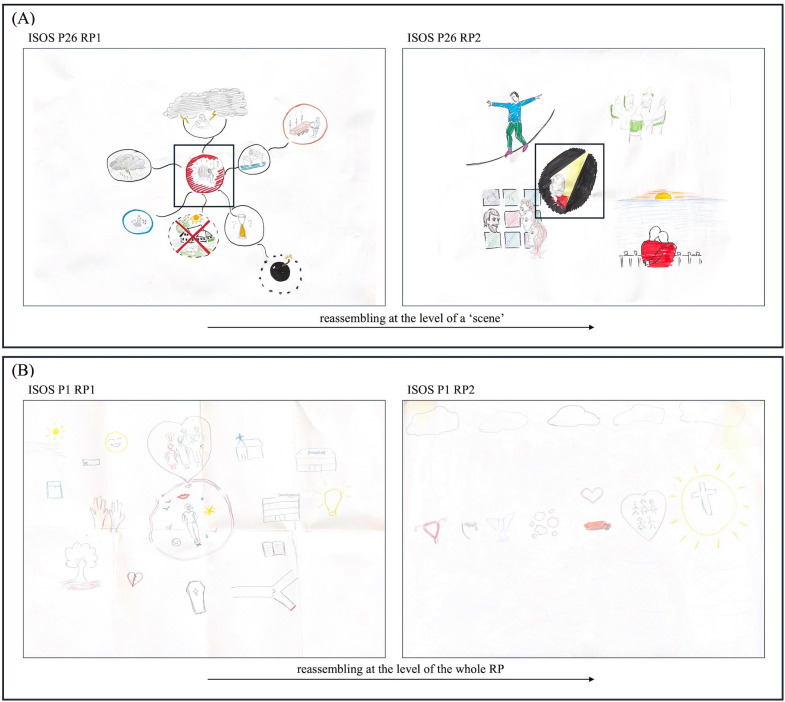
Rich Pictures exemplifying the strategy of reassembly. **(A)** ISOS P26’s RPs exemplifying the strategy of reassembling at the level of a RP scene. * This can be seen in the central scene of both RP1 and RP2 depicting ISOS P26 receiving a phone call about their diagnosis. Here, ISOS P26 repositions themselves from facing away in RP1, to facing forward in RP2. **(B)** ISOS P1’s RPs exemplifying the strategy of reassembling at the level of the whole RP. This can be seen as the scattered and largely disconnected components of RP1 are re-ordered in RP2 to have more compositional linearity and progression (see the linear formation of components within the centre of the RP, reading from left to right), and are framed as/by depictions of flow (see clouds above and flows of water or wind below). * These RPs, without amendments indicating strategies, were published before under the terms of the Creative Commons Attribution License (CC BY 4.0) [26].

The final strategy observed when comparing the RPs was ‘reassembly’ – the structural reorganisation and re-composition of (aspects) of the RP. Though this strategy could also be seen as complementary to other strategies – for example, as participants foregrounded and backgrounded aspects towards reinforcing and reducing – reassembling came to denote the more general and distinct reconfiguration of aspects of or entire RPs by participants. For instance, ISOS P26 (see Fig 4A) depicted the same moment of receiving news about their illness over the phone in both RPs; yet, in doing so, repositioned himself from *facing away* surrounded by the colour red (RP1), to *facing forwards* in a stream of light (RP2). In doing so, one could see that the shift was not (only) in emphasis, but rather, in configuration, perspective and positioning within, and of, the narrative.

Whilst ISOS P26 examples the reassembly of specific scenes within an RP, the strategy of reassembly was also, and strikingly, seen at the level of the entire RP as all motifs were re-ordered in relation to one another (see Fig 4B). Notably, this reassembly of visual narratives appeared as a shift from “muddled” (GW1 P3) or disordered narratives – showing visually scattered or disconnected components – towards ordered narratives, characterised by compositional linearity, progression or flow. This was remarked upon as a kind of “sorting” process (GW1 P3), distinctively exampled in ISOS P1 RP1 and RP2 (Fig 4B):

GW2 P2: There is more order [...] This is drawn in a very orderly manner, so maybe there is more peace in their mind to put things in order.

The strategy of reassembly was used predominantly by ISOS participants. Few NI participants were able to make minor adjustments to their configuration, and no NI participants were seen to reorder narratives in the way exampled by ISOS P1.

## Discussion

This is the first study to examine how individuals construct and reconstruct narrative meaning over time in the context of supportive cancer care using repeated RPs. People with incurable cancer included in our study used six strategies for the reconstruction of their narratives over time: (1) repeating – re-presenting the same, unchanged visual narrative; (2) retaining –keeping aspects of one visual narratives in the next; (3) repurposing – adapting or transforming previously used narrative aspects; (4) reinforcing – intensifying aspects of their narratives; (5) reducing – the shrinkage, removal or refinement of narrative aspects; and finally (6) reassembling – the reconfiguration of aspects of, or the whole, RP. In doing so, participants could be seen to variably work to negotiate – often coexisting – notions of narrative disruption, suspension, continuity, and change, highlighting the complex, and dynamic nature of the evolution of visual illness narratives over time. In this way, our strategies are best understood as theoretically distinct types, which in practice often coincided and overlapped.

Some similar strategies, such as the use of existing, new, strengthened, or adapted meaning-making sources, have been discussed in meaning-making research with people during the first 18 months following curative cancer treatment [38]. Interestingly, this similarity in some strategies suggests that they may be more broadly applicable for supporting different groups in making meaning of illness experiences. We add further strategies to those delineated by Visser et al. [38], and draw them into a narrative meaning-making framework.

Moreover, the employment of these strategies was found to differ between participants undergoing arts-based intervention (ISOS) and no intervention (NI). ISOS participants were predominantly able to repurpose, reinforce, and – sometimes drastically – reassemble aspects of their narratives. They were also able to reduce and remove disruption, as well as intensify and rearrange their narratives towards themes of progression and flow. Comparatively, NI participants had a continued emphasis on retaining aspects of their narratives – particularly medical and somatic experiences – and retaining, reinforcing, and not removing the theme of disruption. They thus appeared to remain engaged in an ongoing narrative negotiation of disruption.

By observing these differences between the groups in both the strategies used and the changes in what they tell, we suggest that our six strategies could be situated, and further investigated, in the process of assuming different narrative modes of relating to contingency – namely, denying, acknowledging, accepting, and receiving [39]. These findings also suggest that arts-based narrative interventions may uniquely support in this process. It has been hypothesized elsewhere that participation in arts-based narrative interventions may afford the so-called ‘narrative aesthetic’ perspective [11]. This is a kind of self-distancing which is afforded through interaction with the arts, that allows for the exploration of new ways to narrate and relate to disruptive, contingent life events [8,11]. Our findings may support this hypothesis.

In addition, our findings showed that differences between an intervention and non-intervention group were not always clearly binary with respect to the use or non-use of strategies. Rather, we found that there was a difference in specific *use* of the strategies, such as the *degree to which* they were employed and *how*. We thus pose these as a means for further nuanced investigation of narrative reconstruction across groups.

By identifying these strategies for narrative reconstruction and highlighting variations in their use, we suggest that the longitudinal use of RPs offers useful insights into narrative meaning-making in the context of illness – over time, across participant groups, and in relation to different interventions.

### Further opportunities and limitations

Durations between RPs in the ISOS and NI studies varied. The longer intervals, as in the ISOS group (median 7 months), may have allowed for more time for reflection and change in contextual circumstances shaping more significant narrative changes or adaptations [12]. Comparatively, shorter intervals, as in the NI group (median 2 months), may have allowed for less time for reflection, less change in context, and thus subtle shifts [12]. Discrepancy in interval durations may therefore have contributed to observed variations in (degree of) narrative strategy use, with ISOS’s longer duration perhaps supporting the development of new ways of narrating and relating to cancer, and NI’s shorter duration contributing to the ongoing narrative negotiation of disruption. This may thus complicate the direct comparisons made between the two groups to indicate the potential support of the intervention in narrative reconstruction. As such, we call for greater understanding of the complex role of time in illness narratives, and how stories about illness evolve over different durations [12]. This would assist in the development of (research methods for) interventions in narrative meaning-making processes. Future studies comparing repeated RPs across groups might therefore take variation in interval duration as an explicit point of enquiry – or otherwise work to ensure that durations are more closely aligned.

Secondly, the ISOS and NI groups varied in relation to one another demographically. Variations included sex, with ISOS participants including more female participants (n = 8/12), and NI participants almost equally including male and female participants; occupational level, with ISOS participants primarily in high skill-level occupations (n = 11/12), and NI participants including both high skill-level and moderate-low skill level; as well as WHO performance status and cancer types. Situating narrative meaning-making within its social and cultural context also suggests that sociodemographic characteristics may play a role in the way people (are able) to reconstruct their stories over time [10]. In addition to this, sociological research suggests that the skill to self-distance (within arts-based interventions) for narrative reconstruction may be socially inculcated – thus, making it more and less available to different sociodemographic groups [19].

This raises the question of whether differences found between ISOS and NI in narrative strategy use over time can be explained, not only by the intervention, but also by differences in sociodemographic characteristics and socially developed skills for narrative reconstruction. For example, the predominance of high skill-level occupations within the ISOS group – one potential indicator of higher socio-economic status – may suggest that they had relative advantages in resources and skills for narrative reconstruction compared to those in lower skill-level occupations in the NI group [10,19]. Moreover, because of the varying degree of representation in popular culture for different cancer types, participants living with breast cancer – more predominant in ISOS – may, for example, have had greater access to cancer-specific narrative resources, both supporting and constraining their narrative reconstruction, than those with less commonly represented cancers (e.g., oesophageal cancer, more predominant in NI). Yet, to understand how stories by different sociodemographic groups evolve, both over time and across arts-based interventions, more explicit research is needed. Future research might investigate these questions by comparing repeated RPs across different sociodemographic groups, without intervention; different groups within the same intervention; and the same sociodemographic group with and without an intervention.

Some further limitations lie in our methods of analyses. Researchers and gallery walk participants were aware which RPs were from patients who had undergone the intervention, potentially biasing judgement/interpretation. We suggest that participants of gallery walks could be blinded regarding the makers of the RPs to help mitigate this [34]. Though we explored a visual analysis of RPs without interviews – and found value in engagement with the RPs themselves – integrating participant interpretations and perspectives may have also enriched findings. Thus, we suggest exploration of approaches wherein participant interpretation and systematic analysis of the RPs themselves are viewed as complementary analytical stages [34]. Lastly, our analyses of RPs were highly time-consuming and laborious, limiting its integration into large-scale studies settings. Improving the efficiency of such analyses, for example through AI, would be an important step in the development of RPs as an evaluative tool for existential interventions [34].

## Conclusion

In this study, we identified six strategies used by people with incurable cancer to evolve their narratives over time. We suggest that these strategies – and their specific application – may serve in the narrative process of assuming different modes of relating to contingency. Arts-based narrative interventions appeared to assist in this process by fostering a narrative-aesthetic perspective, enabling individuals to explore new ways to narrate and relate to the diagnosis of cancer as a disruptive, contingent life event.

The repeated use of RPs provided useful insights into the existential process of narrative meaning-making in illness over time, across groups, and in response to an existential care intervention. These insights demonstrate the potential of repeated RP use to evaluate the impact of such interventions and to inform the development of effective existential supportive care within oncology and broader clinical settings.

## Supporting information

S1 Fig Inclusion of participants in Drawing Cancer Chronicles. This flowchart illustrates the inclusion process for ISOS and NI participants in the DCC analyses based on the described inclusion and exclusion criteria. All those excluded from (specific) DCC analyses are highlighted on the right of the figure with reasons for exclusion. Boxes in bold highlight participants included in (specific) DCC analyses.
(TIF)

S1 Table ISOS and NI participant demographics. (DOCX)

S1 File Example RPs. (PDF)
