# Supplementary material for: Drawing cancer chronicles: A qualitative study to evaluate narrative meaning-making over time and in response to a meaning-centred care intervention
Source: PLoS One. 2026 Jan 20;21(1):e0341150. doi: 10.1371/journal.pone.0341150 (PMC12818685; doi:10.1371/journal.pone.0341150)
Supplement: S1 Table — (DOCX) [file pone.0341150.s002.docx]

|  | ISOS total and NI with 2 RPs | ISOS total | NI with 2 RPs | ISOS with 2 RPs | ISOS and NI with 2 RPs |
| --- | --- | --- | --- | --- | --- |
|  | **n = 38** | **n= 25** | **n = 13** | **n = 12** | **n = 25** |
| Characteristic | Frequency (%) |  |  |  |  |
| Sex |  |  |  |  |  |
| Female | 24 (63.2%) | 18 (72.0%) | 6 (46.2%) | 8 (66.7%) | 14 (56.0%) |
| Male | 14 (36.8%) | 7 (28.0%) | 7 (53.8%) | 4 (33.3%) | 11 (44.0%) |
| Age in years, median (range) | 60 (28-80) | 59 (28-80) | 64 (31-79) | 55.5 (43-80) | 60 (31-80) |
| Type of cancer |  |  |  |  |  |
| Esophageal | 9 (23.7%) | 4 (16.0%) | 5 (38.5%) | 2 (16.7%) | 7 (28.0%) |
| Breast | 6 (15.8%) | 6 (24.0%) | 0 | 3 (25.0%) | 3 (12.0%) |
| Pancreatic | 6 (15.8%) | 2 (8.0%) | 4 (30.8%) | 1 (8.3%) | 5 (20.0%) |
| Gastric | 5 (13.2%) | 2 (8.0%) | 3 (23.1%) | 0 | 3 (12.0%) |
| Cervical | 3 (7.9%) | 3 (12.0%) | 0 | 2 (16.7%) | 2 (8.0%) |
| Colon | 2 (5.3%) | 1 (4.0%) | 1 (7.7%) | 0 | 1 (4.0%) |
| Bile duct | 2 (5.3%) | 2 (8.0%) | 0 | 2 (16.7%) | 2 (8.0%) |
| Rectal | 1 (2.6%) | 1 (4.0%) | 0 | 0 | 0 |
| Neck | 1 (2.6%) | 1 (4.0%) | 0 | 0 | 0 |
| Endocrine | 1 (2.6%) | 1 (4.0%) | 0 | 1 (8.3%) | 1 (4.0%) |
| Bone | 1 (2.6%) | 1 (4.0%) | 0 | 1 (8.3%) | 1 (4.0%) |
| Sarcoma | 1 (2.6%) | 1 (4.0%) | 0 | 0 | 0 |
| WHO performance status at RP1 |  |  |  |  |  |
| 0 | 20 (52.6%) | 14 (56.0%) | 6 (46.2%) | 8 (66.7%) | 14 (56.0%) |
| 1 | 15 (39.5%) | 10 (40.0%) | 5 (38.5%) | 4 (33.3%) | 9 (36.0%) |
| 2 | 2 (5.3%) | 1 (4.0%) | 1 (7.7%) | 0 | 1 (4.0%) |
| Unknown | 1 (2.6%) | 0 | 1 (7.7%) | 0 | 1 (4.0%) |
| WHO performance status at RP2 |  |  |  |  |  |
| 0 | - | - | 7 (53.0%) | 6 (50.0%) | 13 (52.0%) |
| 1 | - | - | 3 (23.1%) | 5 (41.7%) | 8 (32.0%) |
| 2 | - | - | 1 (7.7%) | 0 | 1 (4.0%) |
| 3 | - | - | 1 (7.7%) | 0 | 1 (4.0%) |
| 4 | - | - | 0 | 1 (8.3%) | 1 (4.0%) |
| Unknown | - | - | 1 (7.7%) | 0 | 1 (4.0%) |
| Change in WHO performance status between RP1 and RP2 |  |  |  |  |  |
| -1 | - | - | 3 (23.1%) | 0 | 3 (12.0%) |
| 0 | - | - | 6 (46.2%) | 10 (83.3%) | 16 (64.0%) |
| 1 | - | - | 2 (15.4%) | 1 (8.3%) | 3 (12.0%) |
| 2 | - | - | 1 (7.7%) | 0 | 1 (4.0%) |
| 4 | - | - | 0 | 1 (8.3%) | 1 (4.0%) |
| Unknown | - | - | 1 (7.7%) | 0 | 1 (4.0%) |
| Educational level |  |  |  |  |  |
| University of Applied Sciences | 13 (34.2%) | 12 (48.0%) | 1 (7.7%) | 4 (33.3%) | 5 (20.0%) |
| Academic | 8 (21.1%) | 7 (28.0%) | 1 (7.7%) | 5 (41.7%) | 6 (24.0%) |
| Unknown | 8 (21.1%) | 0 | 8 (61.5%) | 0 | 8 (32.0%) |
| Vocational | 6 (15.8%) | 4 (16.0%) | 2 (15.4%) | 2 (16.7%) | 4 (16.0%) |
| Pre-vocational | 3 (7.9%) | 2 (8.0%) | 1 (7.7%) | 1 (8.3%) | 2 (8.0%) |
| ISCO-08 Major Group categorisation |  |  |  |  |  |
| 1 Managers | 8 (21.1%) | 6 (24.0%) | 2 (15.4%) | 4 (33.3%) | 6 (24.0%) |
| 2 Professionals | 18 (47.4%) | 13 (52.0%) | 5 (38.5%) | 7 (58.3%) | 12 (48.0%) |
| 3 Technicians and Associate Professionals | 1 (2.6%) | 1 (4.0%) | 0 | 0 | 0 |
| 4 Clerical Support Workers | 1 (2.6%) | 1 (4.0%) | 0 | 1 (8.3%) | 1 (4.0%) |
| 5 Service and Sales Workers | 2 (5.3%) | 2 (8.0%) | 0 | 0 | 0 |
| 6 Skilled Agricultural, Forestry and Fishery Workers | 1 (2.6%) | 0 | 1 (7.7%) | 0 | 1 (4.0%) |
| 7 Craft and Related Trades Workers | 2 (5.3%) | 0 | 2 (15.4%) | 0 | 2 (8.0%) |
| 8 Plant and Machine Operators, and Assemblers | 1 (2.3%) | 0 | 1 (7.7%) | 0 | 1 (4.0%) |
| 9 Elementary Occupations | 0 | 0 | 0 | 0 | 0 |
| 0 Armed Forces Occupations | 0 | 0 | 0 | 0 | 0 |
| Unknown | 4 (10.5%) | 2 (8.0%) | 2 (15.4%) | 0 | 2 (8.0%) |
| Time in months between x and RP1, median (range) |  |  |  |  |  |
| Diagnosis of advanced cancer | 12.5 (2-86) | 9 (3-86) | 21 (2-45) | 10.5 (3-35) | 13 (2-45) |
| Most recent disease progression* | 2 (0-31) | 1.5 (0-31) | 9 (2-28) | 3 (0-15) | 5 (0-28) |
| Time in months between x and RP2, median (range) |  |  |  |  |  |
| RP1 | - | - | 2 (1-3) | 7 (4-12) | 3 (1-12) |
| Diagnosis of advanced cancer | - | - | 22 (4-48) | 20 (9-42) | 21 (4-48) |
| * Participants with additional progression dates – columns from left to right: n=17/38 (44.7%), n = 14/25 (56.0%), n=3/13 (23.1%), n=6/12 (50.0%), n=9/25 (36.0%) | | | | | |
