# Supplementary figures and images for: Drawing cancer chronicles: A qualitative study to evaluate narrative meaning-making over time and in response to a meaning-centred care intervention
Source: PLoS One. 2026 Jan 20;21(1):e0341150. doi: 10.1371/journal.pone.0341150 (PMC12818685; doi:10.1371/journal.pone.0341150)

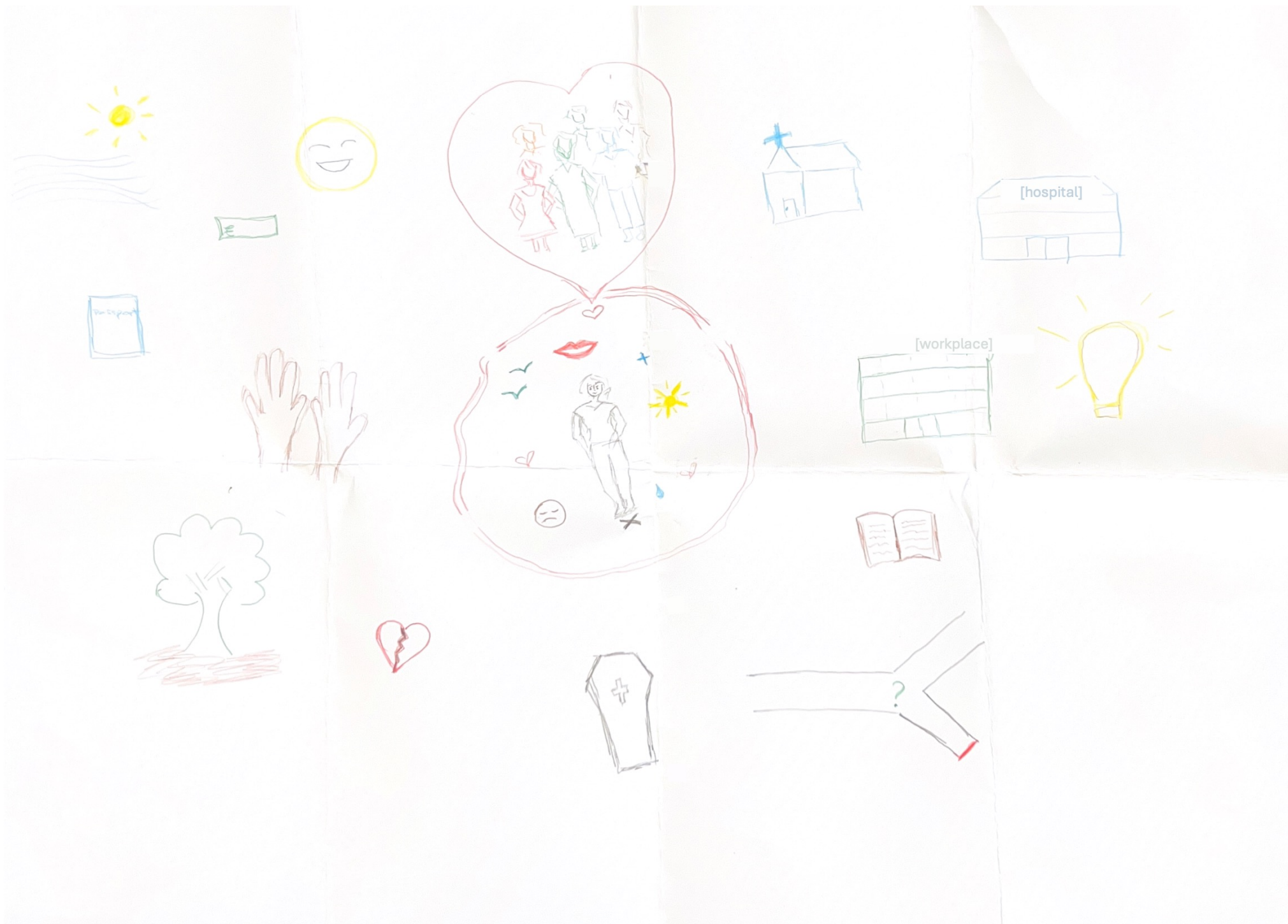

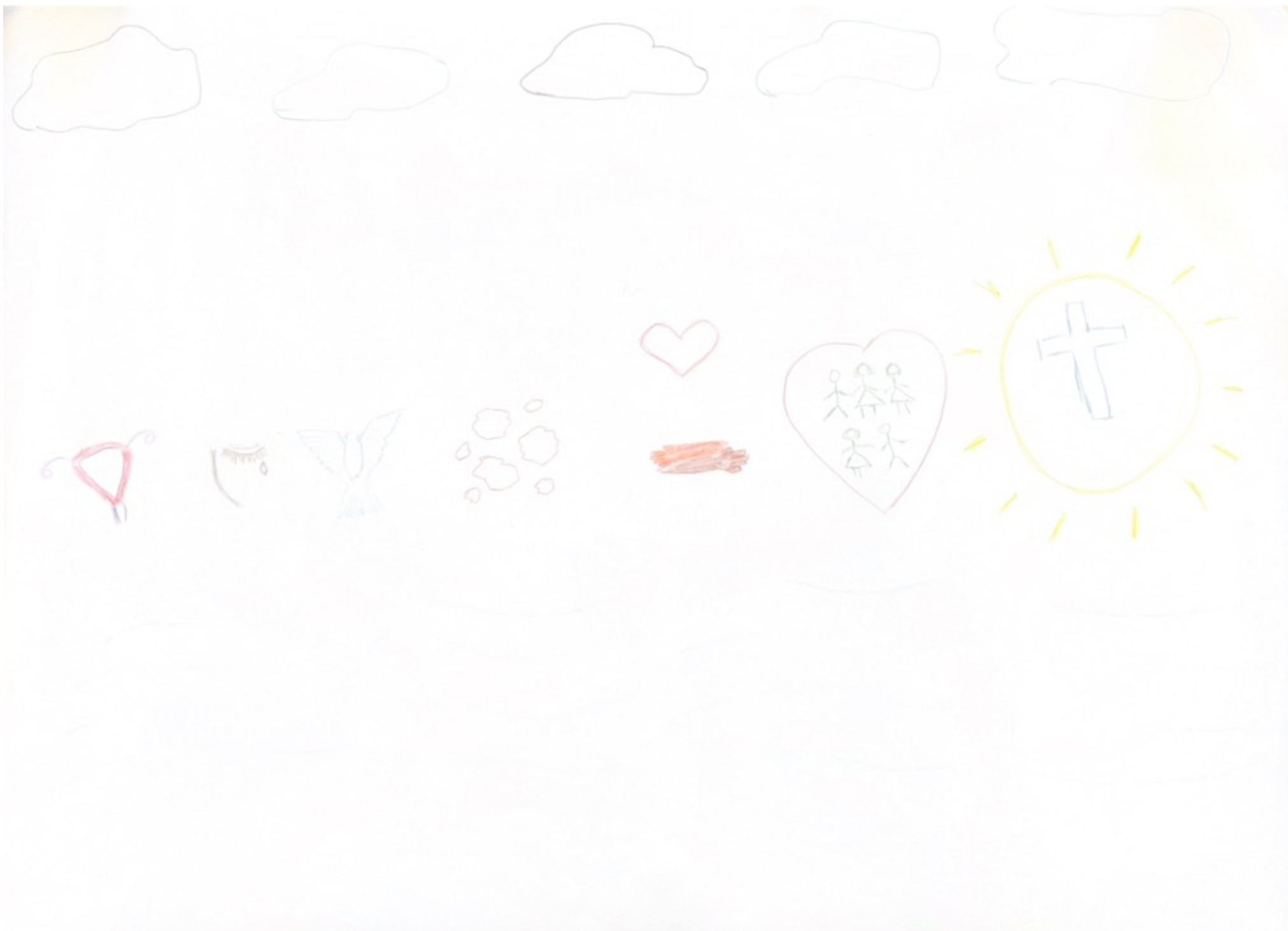

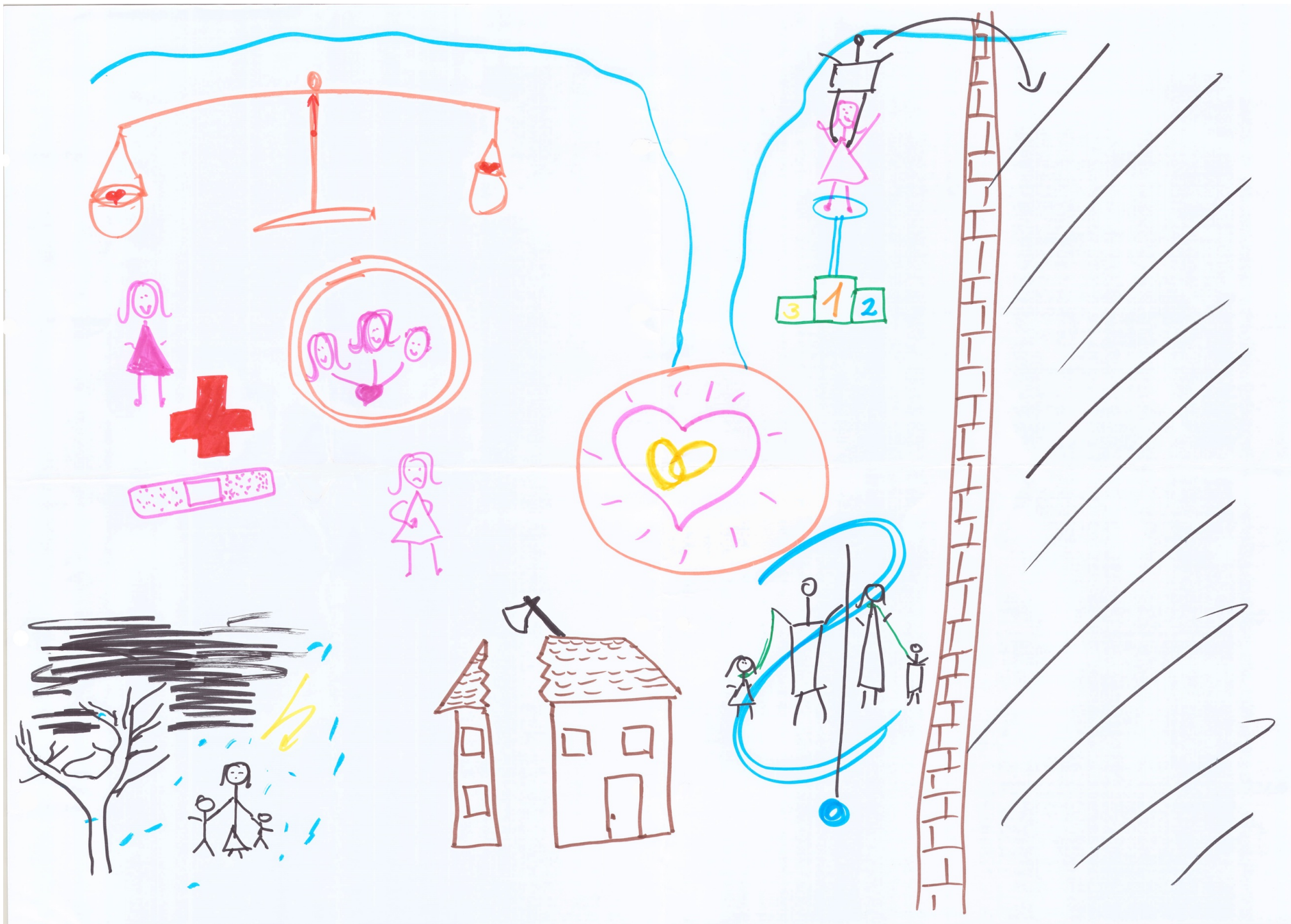

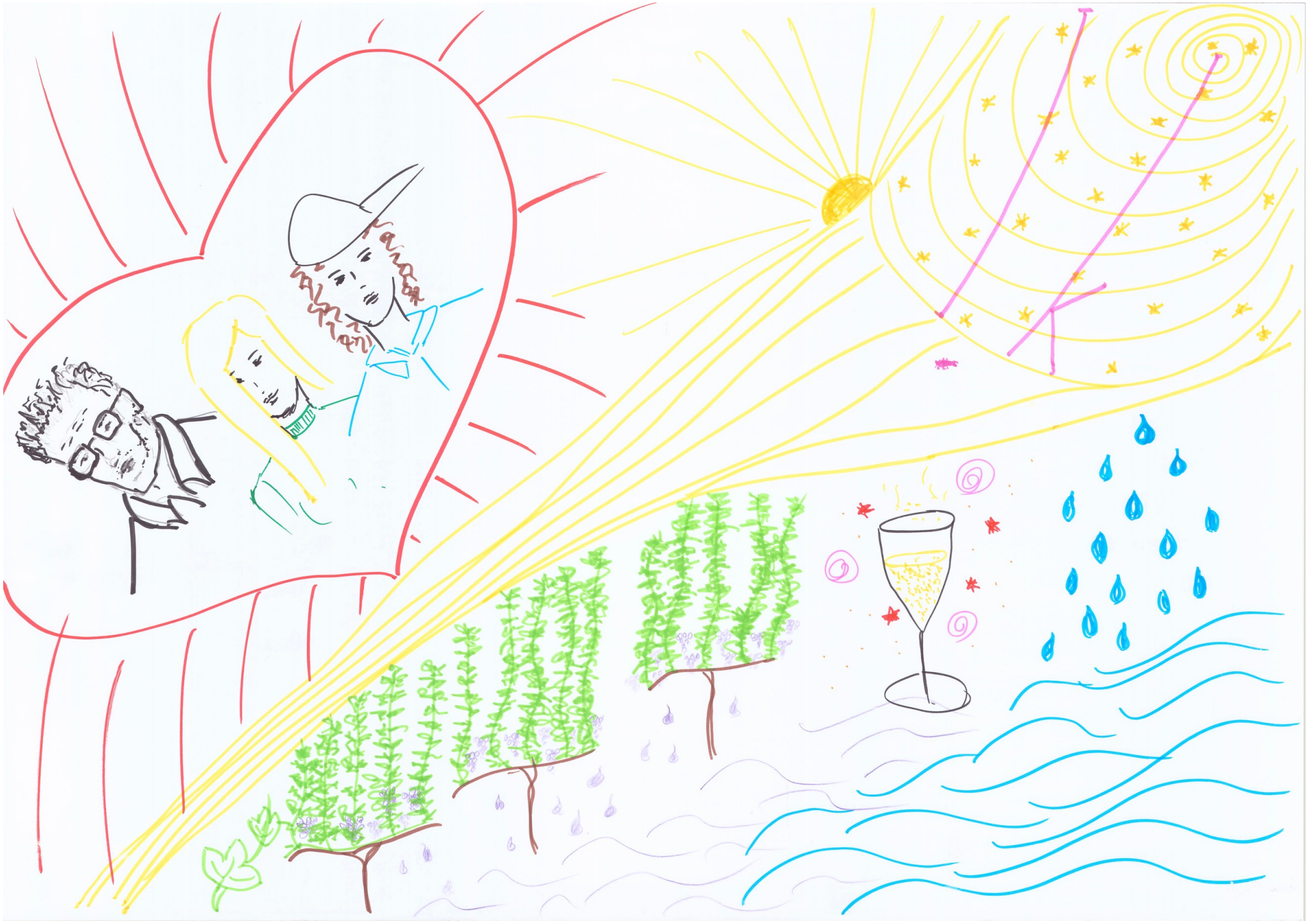

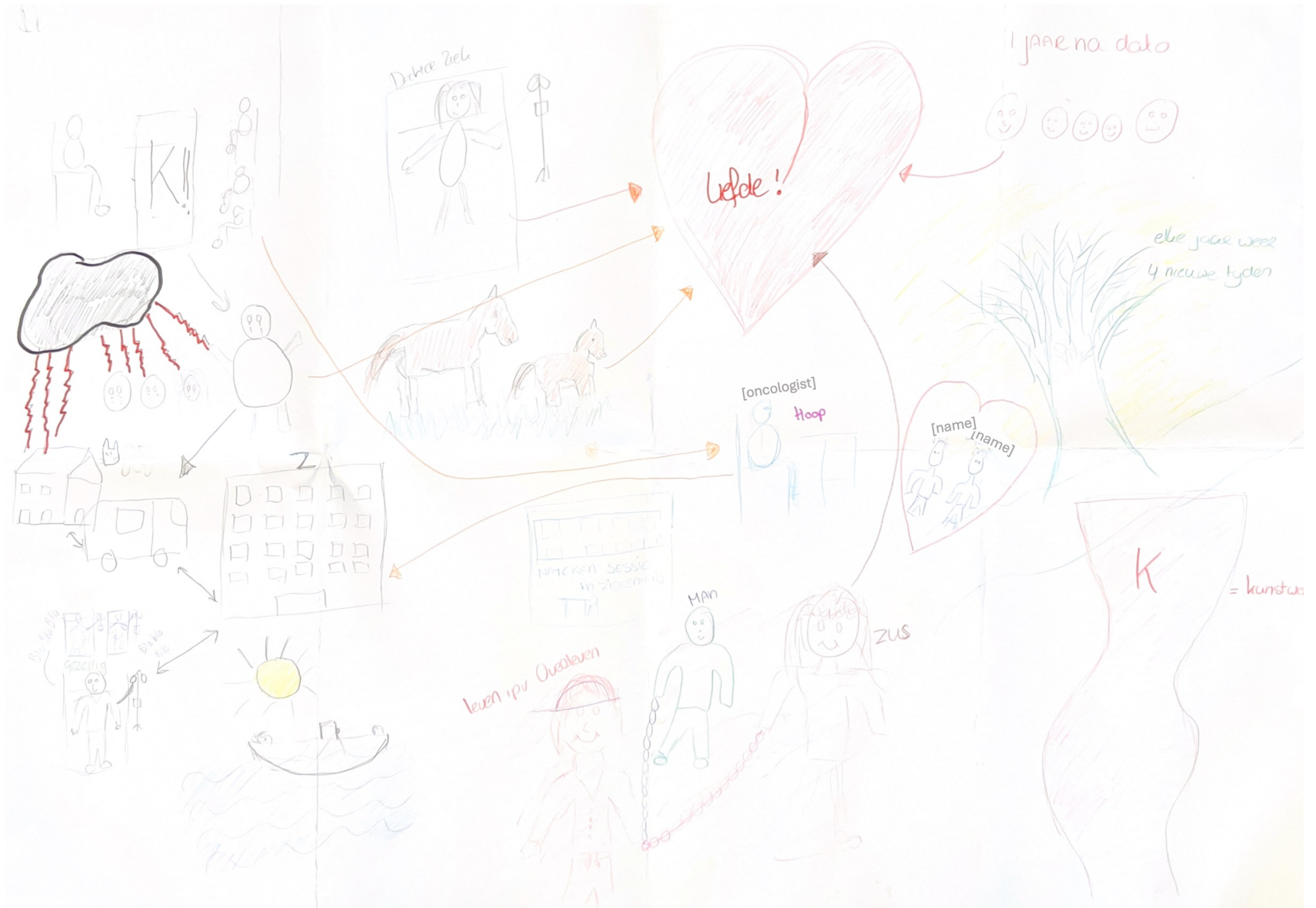

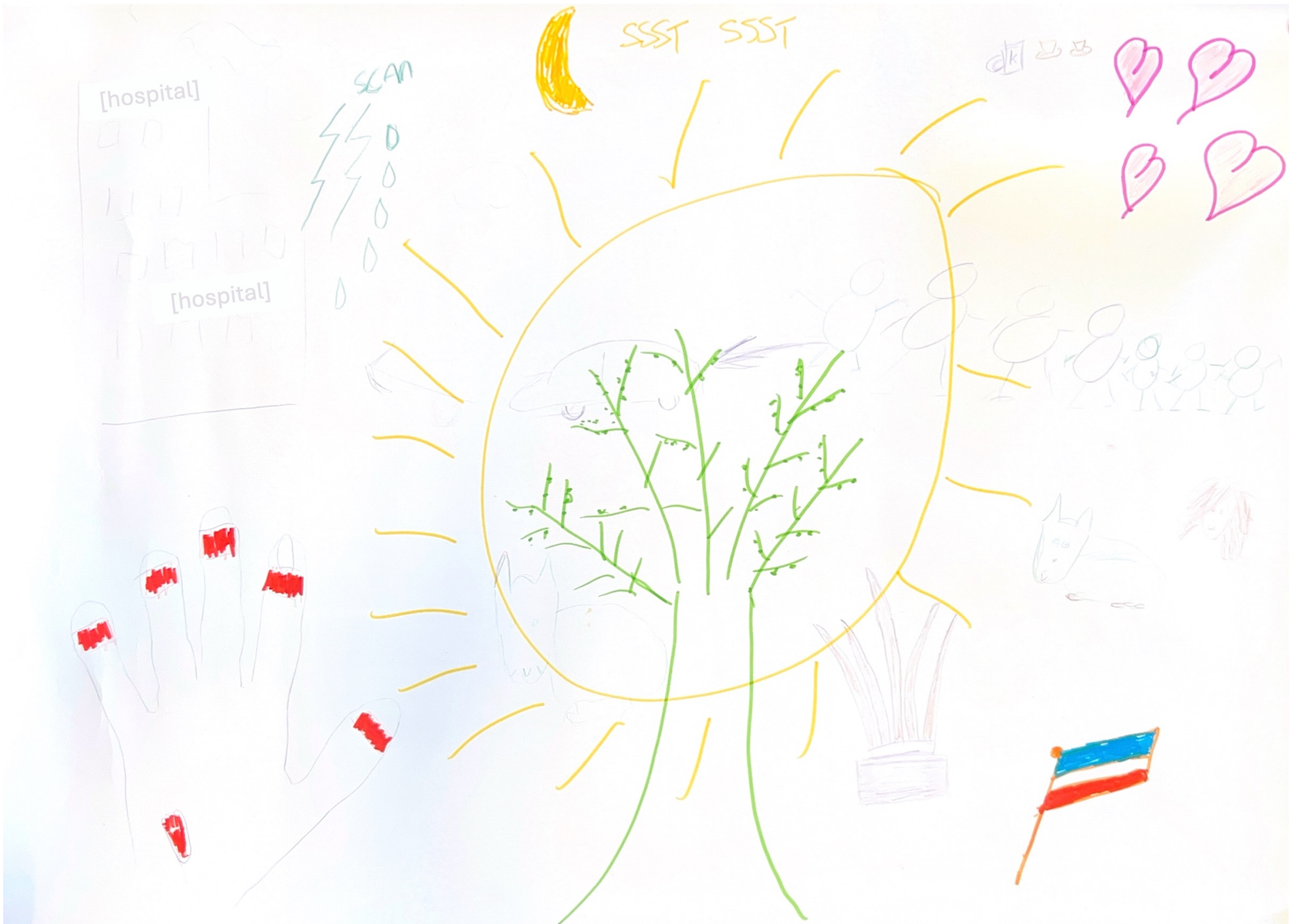

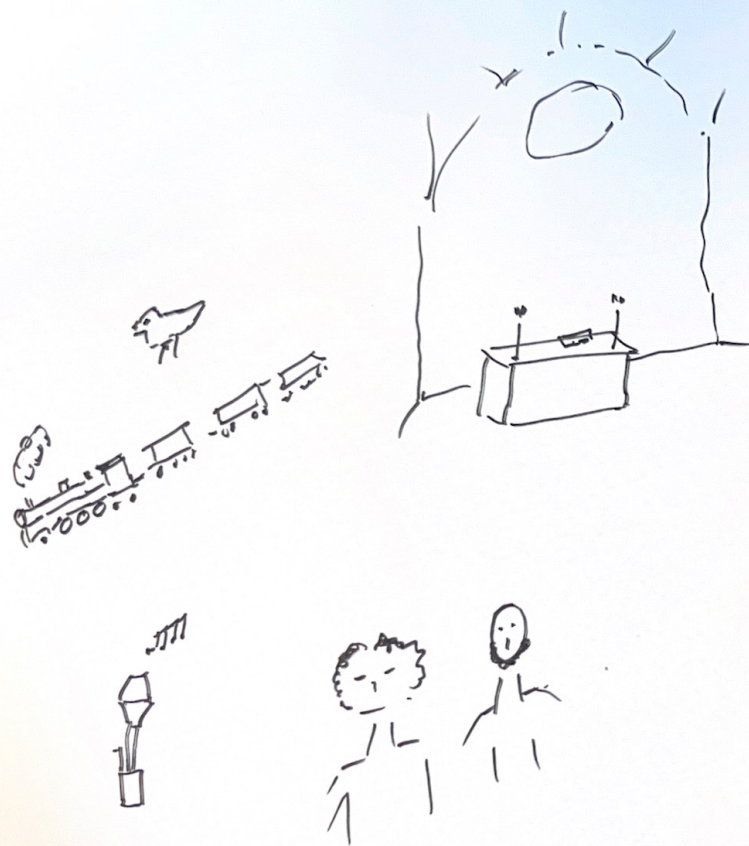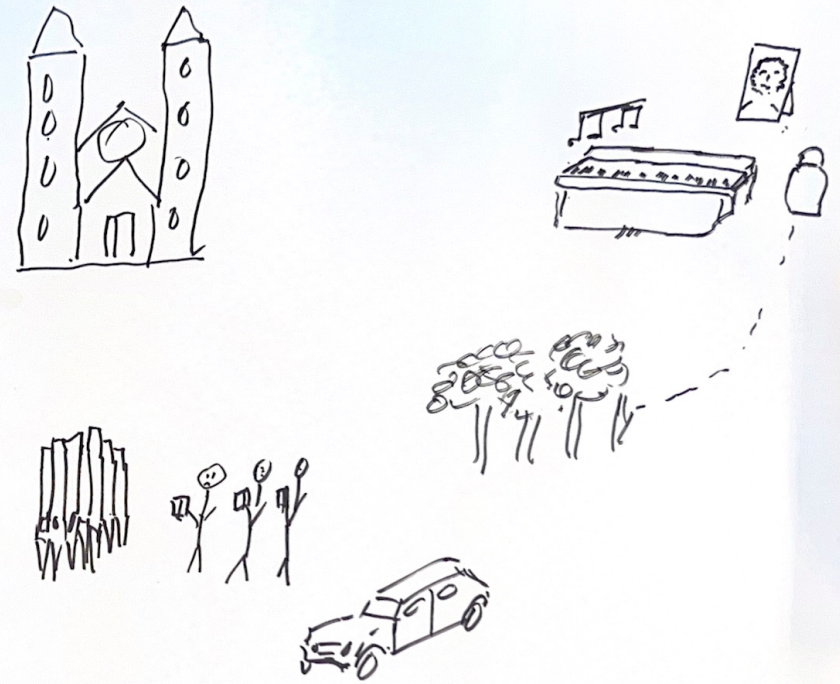

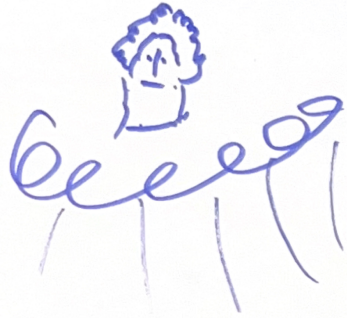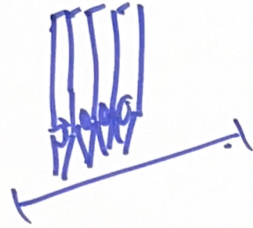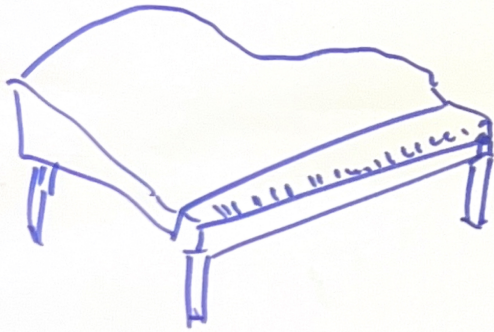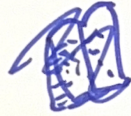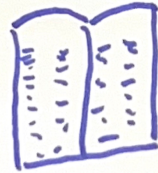

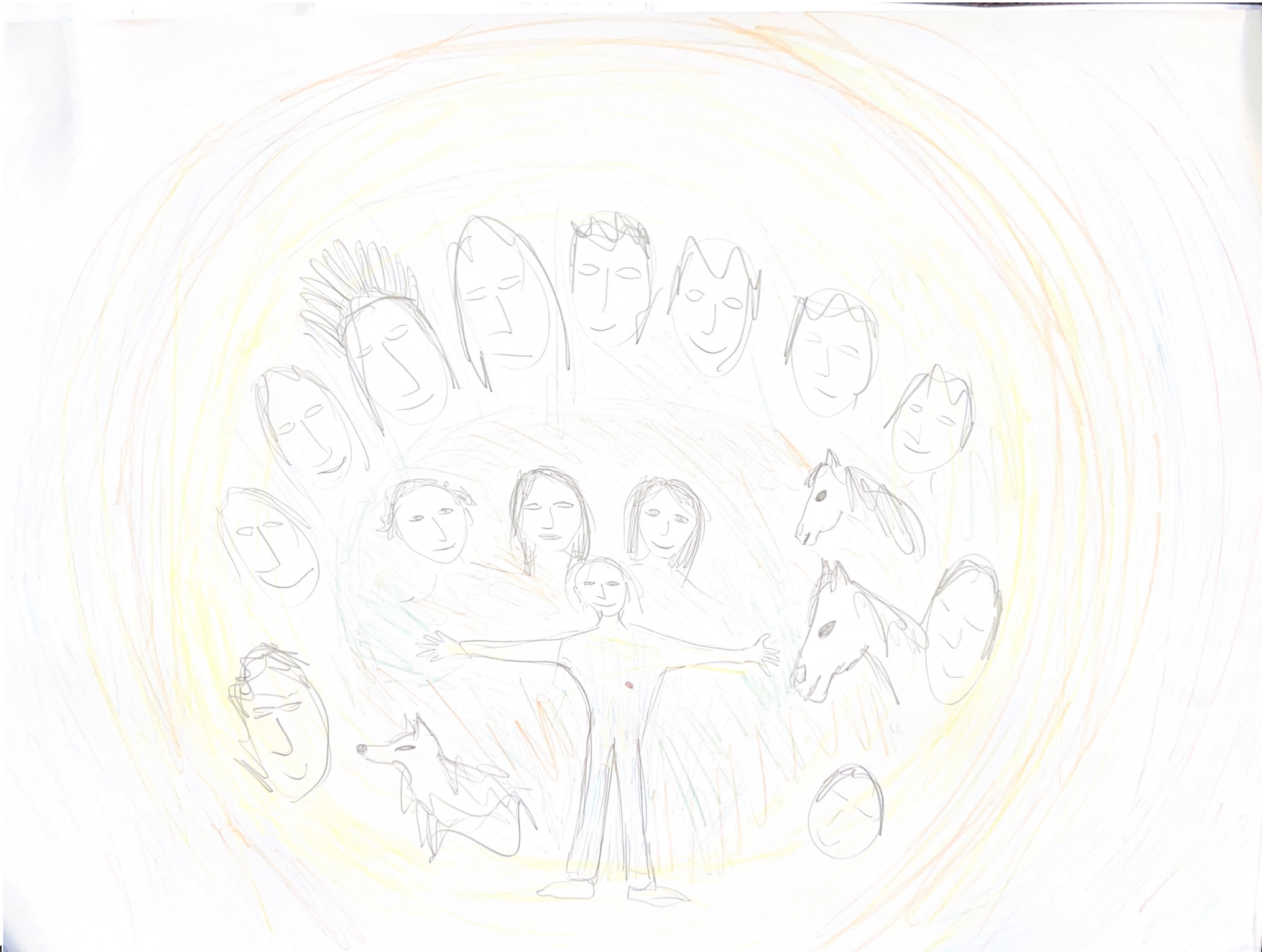

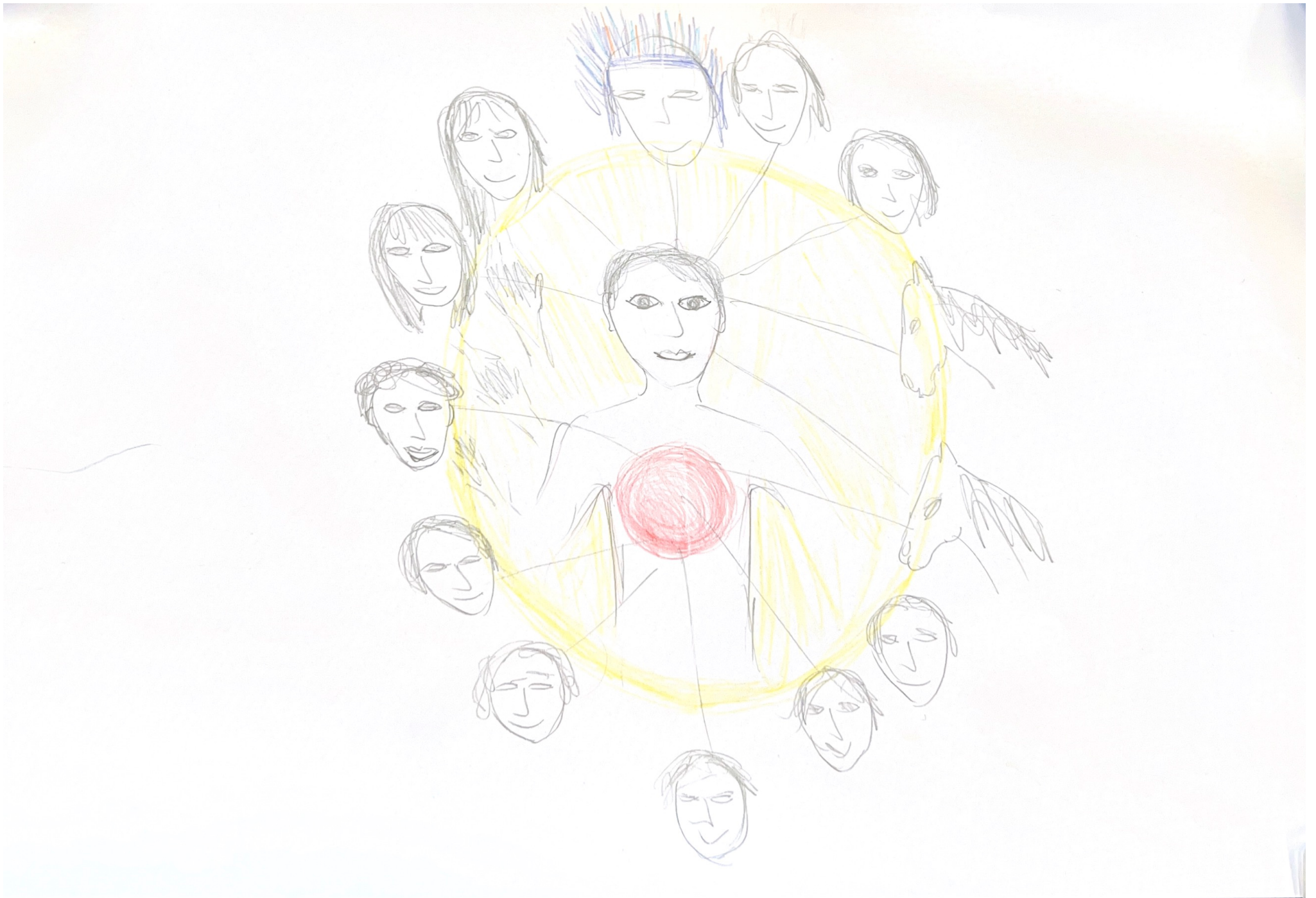

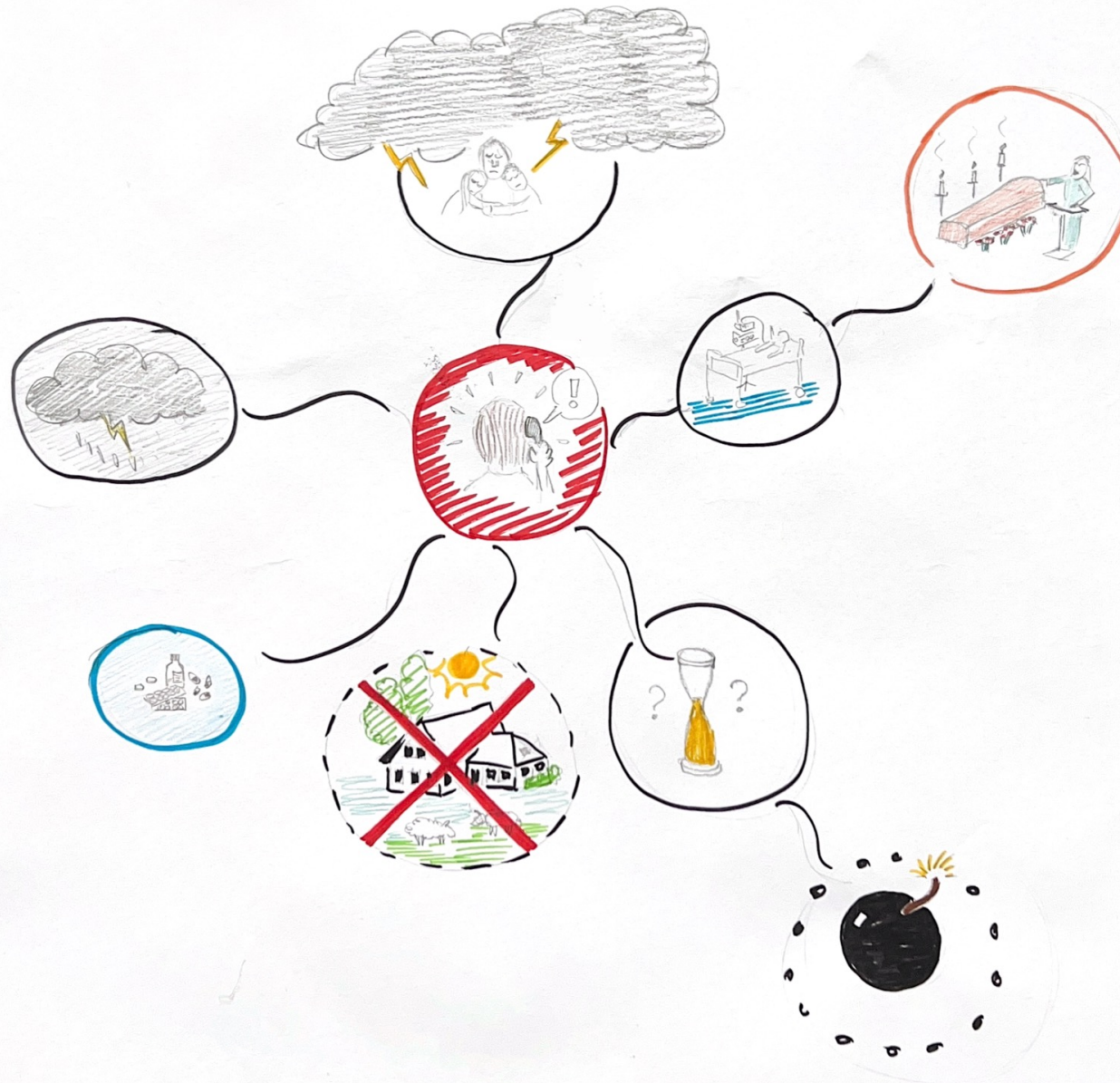

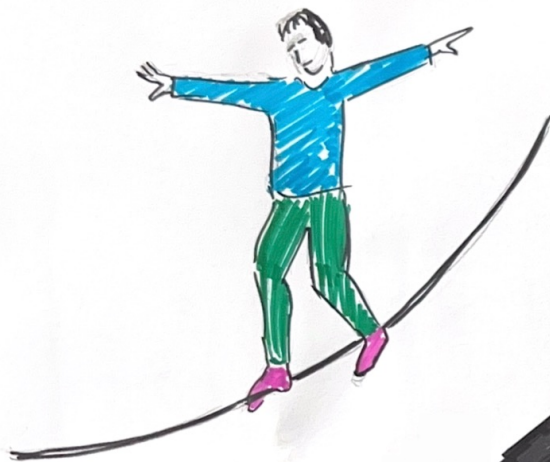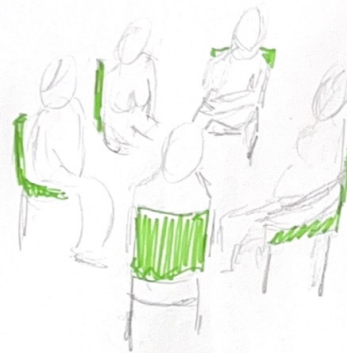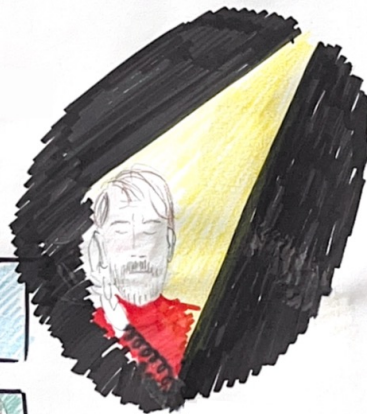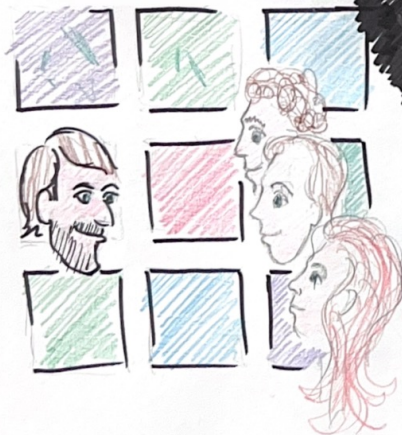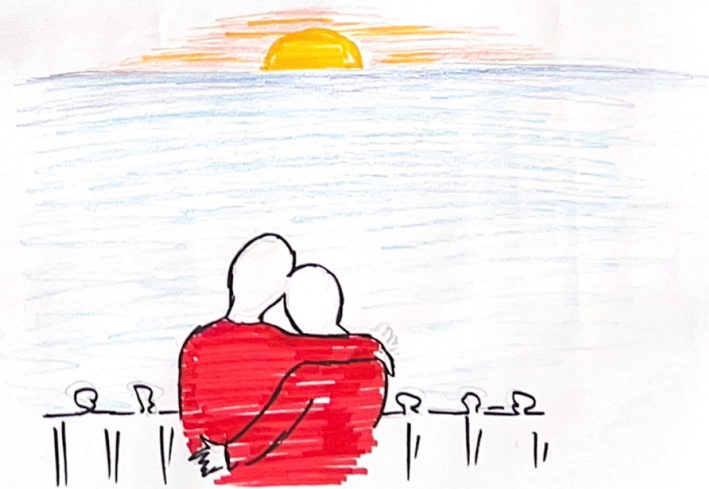

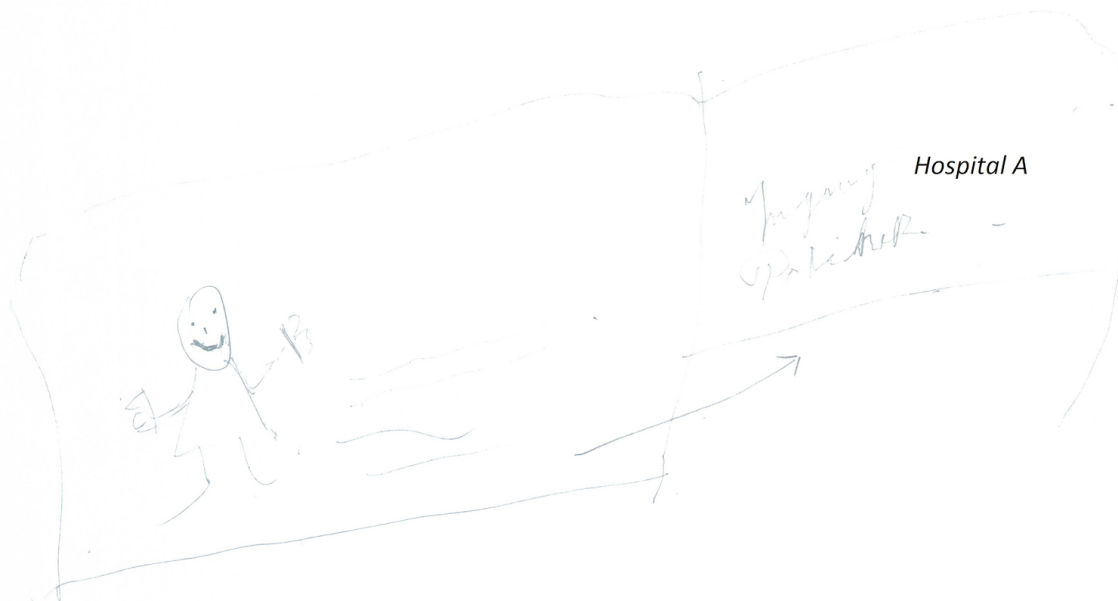

Supplement: S1 File — (PDF) [file pone.0341150.s003.pdf]
